# Exploring perspectives of research ethics committee members on the governance of big data in sub-Saharan Africa

**DOI:** 10.17159/sajs.2023/14905

**Published:** 2023-05-30

**Authors:** Nezerith Cengiz, Siti M. Kabanda, Tonya M. Esterhuizen, Keymanthri Moodley

**Affiliations:** 1 Centre for Medical Ethics and Law, Faculty of Medicine and Health Sciences, Stellenbosch University, Cape Town, South Africa; 2 Division of Epidemiology and Biostatistics, Faculty of Medicine and Health Sciences, Stellenbosch University, Cape Town, South Africa

**Keywords:** big data, data governance, data regulation, research ethics committees, sub-Saharan Africa

## Abstract

Interest in the governance of big data is growing exponentially. However, finding the right balance between making large volumes of data accessible, and safeguarding privacy, preventing data misuse, determining authorship and protecting intellectual property remain challenging. In sub-Saharan Africa (SSA), research ethics committees (RECs) play an important role in reviewing data-intense research protocols. However, this regulatory role must be embedded in a context of robust governance. There is currently a paucity of published literature on how big data are regulated in SSA and if the capacity to review protocols is sufficient. The aim of this study was to provide a broad overview of REC members’ awareness and perceptions of big data governance in SSA. A descriptive cross-sectional survey was conducted from April to July 2022. We invited 300 REC members to participate in our online survey via Research Electronic Data Capture (REDCap). A total of 140 REC members, representing 34 SSA countries, completed the online survey. Awareness of data governance laws, policies and guidelines was variable across the subcontinent. A quarter of respondents (25%) indicated that national regulations on the trans-border flow of research data are inadequate. Institutional policies on research data protection were also regarded as being inadequate. Most respondents (64%) believed that they lacked experience in reviewing data-intense protocols. Data governance and regulation in SSA need to be strengthened at both national and institutional levels. There is a strong need for capacity development in the review of data-intense research protocols on the subcontinent.

## Background

The abundance of health and research data that exists today has enormous potential to unlock future advances in science – a prospect discussed for decades by researchers and policymakers.^1^ Recently, the potential of big data to solve some of the world’s most challenging problems has become more apparent. ‘Big data’ refers to large volumes of a variety of raw data processed at high speed and frequency.^2^ The sharing of research data is of increasing interest, with many funders advocating for, or even requiring researchers to share data sets as a condition of funding to maximise their utility and value.^3^ Understandably, sharing research data is regarded as a best practice by the World Health Organization (WHO).^4,5^

Despite the benefits of data sharing, finding the right balance between making data accessible and safeguarding privacy, preventing data misuse, determining authorship and protecting intellectual property is challenging.^4,6,7^ This challenge has been reported to be greater in low- and middle-income countries (LMICs) such as in sub-Saharan Africa (SSA) because of the gap that exists in decision-making between data producers and data users.^4,7^ Some SSA countries have introduced data protection regulations in response to the recent digital revolution.

South Africa is one of the countries that has sought to enforce data governance via the *Protection of Personal Information Act (POPIA), Act No. 4 of 2013*, which came into force on 1 July 2020.^8^ However, legal and ethics frameworks to guide data sharing and protect the interests of data donors on the subcontinent appear to vary considerably in their structure, terms, procedures and authority.^9^

Data protection has also become concerning in the context of the cross-border transfer of human biological materials (HBMs) and data.^10^ In response to this, Material Transfer Agreements (MTAs) and Data Transfer Agreements (DTAs) have evolved to contractually govern the transfer of biological materials and data between parties to protect the interests of stakeholders.^11^ A DTA is a legal contract governing the transfer of deidentified human subject data, or identifiable human subject data in cases where a respondent has given voluntary, informed and electronic consent.^12^ DTAs are required when data owned by one institution are transferred to another institution for the continuation of research efforts. A DTA sets out the related protection, rights and obligations of both parties and delineates the specific purpose(s) for which the data may be used. This facilitates the cross-border transfer of data.^11,12^ In some countries, there is an additional requirement to inform the relevant national data protection authority about the cross-border transfer of data.

Research ethics committees (RECs) have traditionally been established to protect the rights of research participants. However, they also play an important role in reviewing data-intense research protocols where data protection and data sharing are important.^13^ The recent pandemic has placed increasing demands on RECs as research engaging with big data and artificial intelligence (AI) was accelerated. Many scholars have been deliberating on the role of RECs in reviewing data-intense research protocols, and have found that developed countries such as Switzerland^2^, the UK^14^ and Australia^15^ lack the expertise or skills to review such studies. Big data research should be differently legislated and considered as it poses greater or unique risks and implications than flows of samples. Conventional informed consent is not ideal for protecting participants in big data research.^2^ Other examples of the implications of big data research include anonymisation, algorithmic bias, data protection, data storage and data reuse. In many countries in SSA, biological samples are regulated in legislation via MTAs and in guidelines.^16^ However, data, and particularly big data, are excluded. The rapid flow of large volumes of data carries benefits to science, but also many risks to personal information protection and governance, and should be regulated.

The data ecosystem is becoming increasingly complex. Apart from RECs, Data Access Committees (DACs) have emerged as another governance mechanism to manage the controlled access of data.^13^ A DAC comprises a group of individuals who have the responsibility of reviewing and assessing research data access requests.^13^ They may serve as part of an REC or may be an independent committee in an institution or country with the aim of promoting the benefits of data access, whilst minimising potential harm to data respondents or donors.^13^

Data governance is understood as the practice of safeguarding valuable information from exploitation, compromise and loss or theft. It is largely executed through regulatory and legal data protection frameworks.^17–19^ These frameworks govern how certain data types are collected, processed and shared. This secures the privacy, availability and integrity of data through frameworks that set out how sensitive data, in particular, and privacy should be managed via the provision of tools and policies that restrict the unauthorised access, use and/or transfer of data.^17–19^ Examples of personal identifiable data include names, photographs, email addresses, bank account details, the Internet Protocol (IP) addresses of personal computers and biometric data.^17^

It is important to note that data protection laws may differ across various countries, thereby causing an inequality and disparity in the degree of data protection. Some of these countries have stricter rules that apply, which may require notification or approval by the data protection authority and/or special conditions, as well as consent from the data subject as a requirement for the cross-border transfer of data.^20^

In South Africa, the National Health Research Ethics Council (NHREC) developed a national guideline, ‘Ethics in Health Research: Principles, Processes and Structures’, in 2015 to ensure that research is conducted responsibly and ethically in South Africa.^21^ The NHREC emphasises the importance of recognising the values, beliefs and attitudes of data donors.^21^

The guidance document recommends the responsible management of data collection, informed consent, the protection of vulnerable populations, the permissible secondary use of data, and the non-maleficent use of genetic and genomic research.^21^ However, these guidelines are not specific to big data collection, and improved recommendations are required to meet international standards of data management.^21,22^

Being cognisant of the challenges in the big data ecosystem in LMICs, we aimed to determine REC members’ perceptions of data governance in SSA and to describe related challenges. This study is part of a bigger project exploring the ethical, legal and social implications of big data and AI in SSA.

To date, there are no published studies from SSA that have explored the perspectives of REC members on data governance or on the review of data-intense research protocols. Consequently, it is unclear how REC members on the subcontinent navigate governance structures and processes, and review such protocols. This study offers a novel contribution to the empirical literature in SSA as it aimed to explore these perspectives.

## Methods

### Study design and sampling

A descriptive cross-sectional survey with both quantitative and qualitative components, involving 140 REC members representing 34 SSA countries, was conducted from April to July 2022. Our aim was to recruit at least one representative from each of the 49 SSA countries. The study population was selected based on membership of a private, institutional or national REC in SSA.

Respondents were invited to participate in an online survey through a web-based application, Research Electronic Data Capture (REDCap). We recruited our sample of REC members through a purposive selection of professional networks of the Stellenbosch University’s Centre for Medical Ethics and Law across SSA, and employed a snowballing technique to recruit further respondents.^23^ All respondents participated in their personal capacities and provided online consent prior to their completion of the survey.

### The survey instruments

The questionnaire was developed based on a review of the literature and consultation with experts in research ethics. A final draft of the questionnaire was developed using REDCap. This online questionnaire was piloted with six REC members from Stellenbosch University to assess its legibility, eliminate ambiguous questions, address repetition and identify any missing information. This was to ensure the face validity of the data collection tool.

The piloted version of the questionnaire consisted of 20 closed-ended questions, of which four were conditional questions that required respondents to meet a certain condition to be asked the following question. These questions were used to establish baseline data regarding the existence of research data-sharing frameworks and guidelines in SSA, the level of awareness of these frameworks and guidelines by REC members, and perspectives regarding existing legal and ethical challenges. In the questionnaire, we distinguished between the institutional and national governance of research data protection and the trans-border flow of research data to take into account the SSA countries without national governance laws. These were divergent across some institutions and countries.

The data collection tool was developed in English and translated into French and Portuguese to cater for Francophone and Lusophone countries.

### Data analysis

Survey responses were exported from REDCap into the Statistical Package for Social Sciences (SPSS) version 28 for analysis. Frequencies and percentages were used to describe responses to the closed questions. A trained researcher analysed the answers from the open-ended questions manually by identifying recurring responses.

### Ethical aspects

Research integrity was maintained throughout the study, and participation in this research remained entirely voluntary. This survey was a minimal-risk study as the questionnaires involved a factual enquiry with educated, empowered respondents who had the full capacity to consent or decline participation. We approached members in their individual capacities, and respondents consented in their personal capacities. Ethics approval was granted by the Health Research Ethics Committee of the Faculty of Medicine and Health Sciences (reference no: N22/03/028) at Stellenbosch University, South Africa.

## Results

### Demographic information

**Figure 1:**
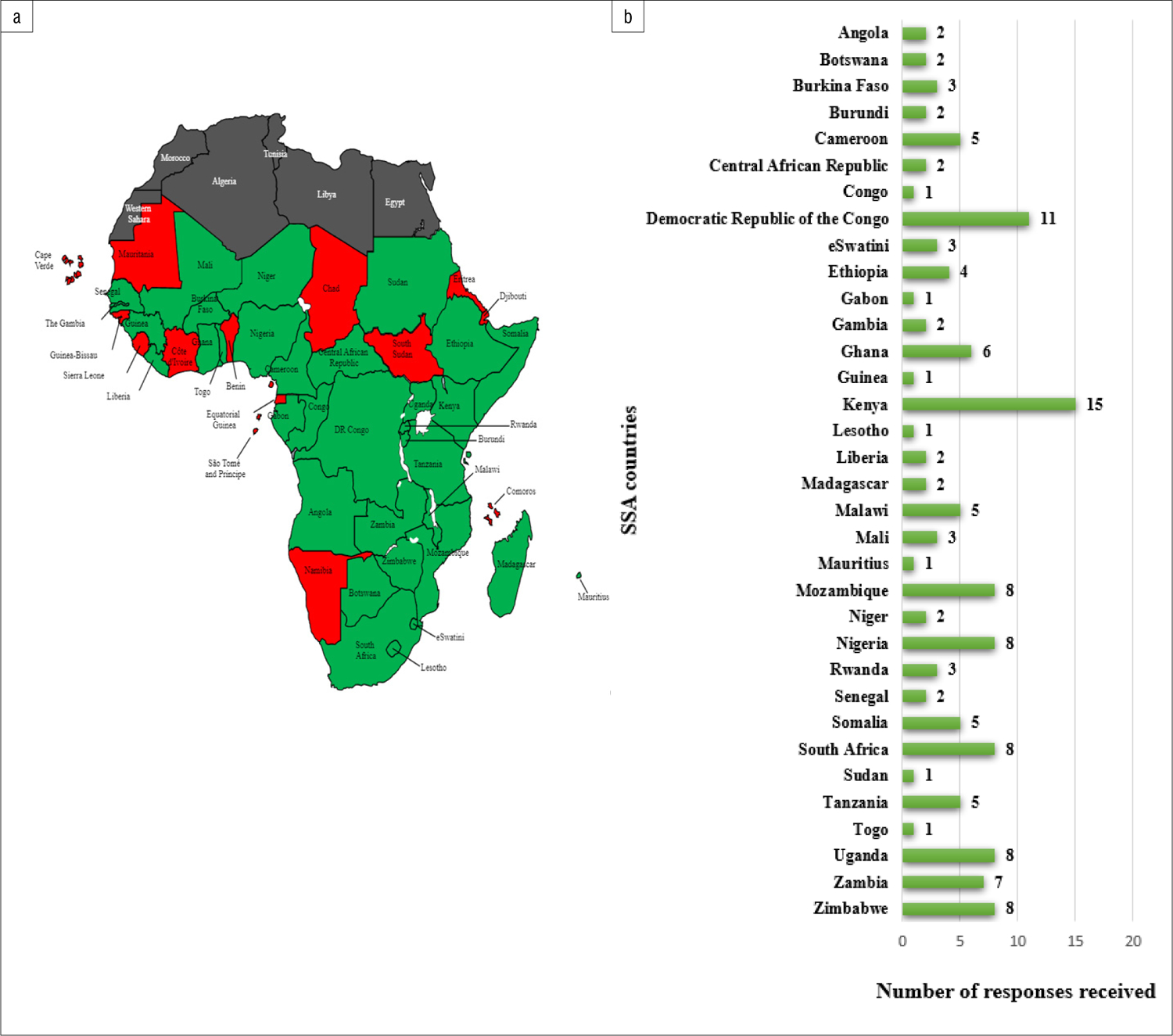
(a) Sub-Saharan Africa (SSA) and (b) the representation of responses received across SSA countries.

A total of 300 individuals were invited to participate in the research study and 140 completed the online survey, yielding an overall response rate of 47% (140/300). The total number of respondents represented 34 of the 49 SSA countries (Figure 1).

More than half the respondents (63%) self-identified as male (88/140), whilst 46% of the respondents (64/140) were PhD graduates, and 41% (58/140) were master’s degree graduates (Table 1). Of the respondents, 80% (112/140) had served in the capacity of an REC member and most responses (69%) came from those who had served on an institutional REC (96/140).

### Awareness of current laws and policies on research data protection

Just over half the respondents (59%; 82/140) indicated that their country had laws on research data protection (Table 2). Less than half (48%; 67/140) indicated that their country had restrictions and/or prohibitions regarding the trans-border flow of research data. We validated whether respondents responded correctly when reporting on the existence of legislation in their respective countries (Table 3). Of 107 respondents, 76% (81/107) showed concordance, whilst 24% (26/107) showed discordance. For this calculation, we excluded the 33 ‘unsure’ responses. The validity, estimated at 76% in the study, was based on this one question.

Most respondents (69%; 96/140) indicated that their institutions had policies on research data protection, and 50% (70/140) specified that restrictions and/or prohibitions for the trans-border flow of research data were also in place. Interestingly, just over a third (34%) of the respondents (48/140) mentioned that their affiliated institutions had no restrictions for the trans-border flow of research data.

### Perceptions of the current laws and policies on research data protection and transfer

Respondents were asked to indicate how much they agreed or disagreed (on a six-point scale) with statements about the adequacy of their country’s laws and institutional policies on research data protection (Table 4). Of the respondents, 45% (63/140) expressed the view that their country’s current laws on research data protection were adequate, whereas 19% (27/140) disagreed. Of those who disagreed, 9% (12/140) disagreed strongly. Similarly, 40% (56/140) of respondents perceived their national restrictions and prohibitions on the trans-border flow of research data to be adequate. Of those who agreed, only 7% (10/140) agreed strongly. Just over half (51%) of all respondents (72/140) perceived their institutional policies on research data protection to be adequate.

On the other hand, a quarter (25%) of the respondents (35/140) indicated that their national restrictions and prohibitions on the trans-border flow of research data were inadequate. Slightly fewer (21%; 29/140) felt that their institutional policies on research data protection were also inadequate.

### Transfer agreements

Awareness of MTAs and DTAs was generally good, but around 20% of respondents (28/140) were uncertain of the existence of such agreements. Just over a third (36%; 50/140) indicated that their institutions had a separate DTA in place. Most respondents (74%; 103/140) indicated that their REC was required to review DTAs and MTAs. Only 13% (18/140) indicated that their REC did not review these documents (Table 5).

Most respondents (64%; 89/140) indicated that they lacked experience in reviewing data-intense protocols that involve data sharing, as up to 50% of all protocols that they reviewed did not relate to data at all, whilst only 14% of respondents (19/140) indicated that more than half of their reviewed protocols related purely to large data sets or big data.

### Support systems for REC members

Respondents were asked to indicate the ease of accessing their country’s data regulatory body for consultation. Over a third (38%) of respondents (53/140) indicated that they could easily do so, whereas 25% (35/140) disagreed. A portion of respondents (12%; 17/140) indicated that no data regulatory body existed within their country.

A minority of respondents 14% (20/140) indicated that they had received no training on how to review protocols involving data sharing. A fifth (21%) of respondents (30/140) indicated that their institution did not have appropriate regulatory policies on the protection of research data and/or HBMs. Likewise, 14% of respondents (19/140) indicated that their institution did not have appropriate ethics guidance on the protection of research data and/or HBMs (Table 4).

### Challenges with data governance

Just over a third (36%) of respondents (51/140) indicated that they faced challenges in their countries regarding the development of legal frameworks or guidance for research data protection. Only 59% of respondents (82/140) reported having current national laws on data protection. The reasons provided were based on poor resources available within these countries, coupled with a lack of capacity to focus on the development of legislation:

The lack of law is the main challenge to be recorded in SSA.

[Country 1]

Specific guidance/law for research data protection is not developed at country level. Laws and [*the*] Constitution address issues related to data protection in fragmented ways.

[Country 2]

Respondents raised a lack of adequately trained legal and ethical experts as another challenge:

The legal experts who develop legal frameworks or guidance for research data protection have not been trained in research ethics. As such, the current legal frameworks for research data protection lack ethical input. Secondly, the current legal frameworks are very restrictive because the regulators are rigid and do not want to move with the signs of the times.

[Country 3]

Lack of legal and ethics experts to develop the frameworks…Lack of trained personnel in this field….

[Country 4]

The lack of awareness regarding research ethics and related issues was raised as an issue:

There is a shortage of knowledge amongst clinician practitioners involved in research requiring the implications of the Protection of Personal Information Act.

[Country 5]

Respondents also identified the lack of clear DTAs for many countries in SSA as a hindrance to good data governance:

We need to come up with a clear DTA.

[Country 6]

Addressing issues related to data in collaborative research. Issues of consent for secondary use of data – use of data for other research not included in the original protocol for which informed consent was provided.

[Country 7]

**Figure 2:**
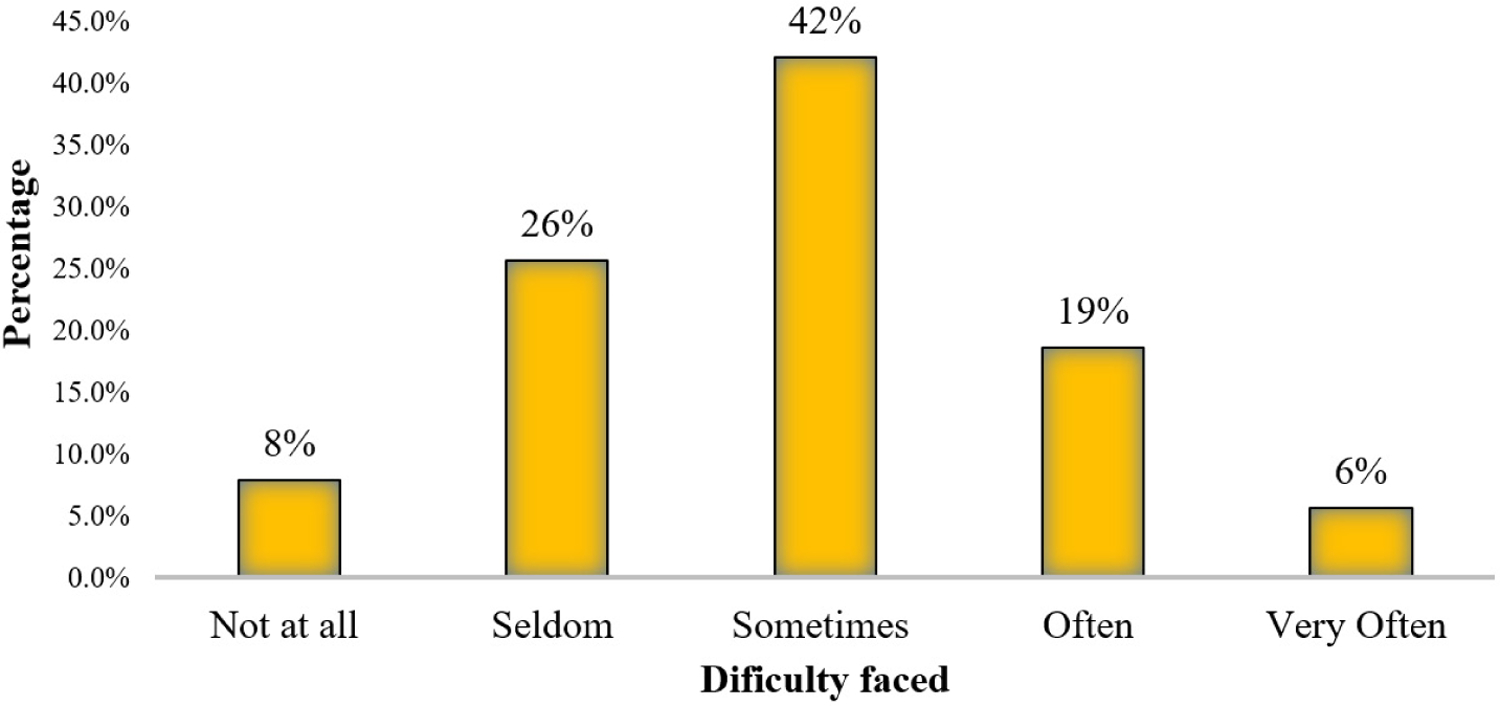
Difficulty in reviewing data-sharing protocols.

The majority of respondents (66%; 93/140) revealed that they experience some level of difficulty in reviewing data sharing related protocols (Figure 2).

### Suggested improvements

Most respondents (71%; 99/140) expressed the view that data sharing for research could be better regulated at their institution. Respondents emphasised a need for the development of institutional policies with clear guidelines for implementation and adequate processes for the follow-up of research protocols. Suggestions around the potential development of DACs within institutions emerged as an idea for the better regulation of data sharing within research.

More than half the respondents (64%; 89/140) indicated that their institutions did not have DACs to handle data-related issues in research. These findings further highlight the need for a DAC as it relates to institutional regulation.

This should start from drafting laws and policies that specifically govern/regulate specimen and data sharing. Research institutions can then draw from these to develop their standard operating procedures or guidelines. External research partners can develop capacity in this area through funding [*the*] training of IRB members involved in the review of protocols that involve samples and data sharing.

[Country 8]

By establishing Data Access Ethics Subcommittees to function under RECs, or better still, provision of training to RECs so that they can play the regulatory role.

[Country 7]

Many respondents suggested the development of comprehensive DTAs to improve regulation at a national level. Qualitative responses highlighted the importance of local and international collaboration and the increased need for support to researchers.

The need to raise awareness through education among research stakeholders, including IRB members, researchers, communities, as well as respondents about the benefits and risks of data sharing. This empowerment will encourage research stakeholders to appreciate the need for [*the*] regulation of samples and data sharing to avoid unethical practices in sample and data sharing like exploitation and harm to individual respondents and communities where the research is conducted.

[Country 8]

We need to support researchers to understand the bigger value of data and appreciate [*the*] value of engaging in data agreements with collaborating institution, which business they have been leaving to the regulator.

[Country 9]

## Discussion

Historically, RECs have been tasked with reviewing classic clinical trials and other research protocols with limited data sets.^24^ Robust governance frameworks exist globally and in SSA to guide this type of research review.^25^ Likewise, a reasonable amount of capacity development has occurred in research ethics review in SSA.^25^ Big data have raised new ethics and legal challenges^26^, and our results provide a broad overview of these challenges in SSA. To our knowledge, this is the first empirical survey in SSA in which awareness and perspectives of REC members have been explored specifically as they relate to the review of data-intense research.

There are governance challenges relating to data protection in research as not all countries in SSA have a legal framework to regulate the use of big data in research. Instead, there is a spectrum of legal regulation, ranging from the strict, comprehensive protection of data to no legal frameworks at all.^27–29^ Likewise, research ethics policies and guidelines suffer the same level of variability across the subcontinent where big data are concerned.^25^

Our study confirms this variability as knowledge and awareness of legislative frameworks and ethics guidance in SSA vary considerably. Only 58% of the REC members surveyed indicated that laws existed at a national level, with the remainder indicating no knowledge or uncertainty about the existence of such laws. More specifically, a quarter (24%) of REC members were uncertain about whether such frameworks existed within their respective countries or institutions.

Most concerning is the apparent lack of legislative frameworks for the cross-border transfer of big data on the subcontinent and out of Africa to other parts of the world. This is important because of the historical concern with data and samples leaving SSA in an unregulated manner, which raises concerns about exploitative research practices.^30–32^ Although just under two-thirds of respondents were unaware of laws relating to data-intense research, only half were aware of laws relating to the cross-border transfer of data. This suggests that research data may be crossing borders without agreements or export permits in place. This is supported by Labuschaigne et al.^33^ who reported that HBMs may be leaving South Africa without export permits or MTAs during collaborative research. Mwaka and Munabi^34^, who undertook a similar study on perceptions and experiences on the transfer of HBMs in international collaborative research in Uganda, reported that the development of an MTA and its implementation lacked transparency.

This concern is reflected at a more granular level as knowledge or awareness of DTAs and DACs demonstrate. Our findings reflect this, as 13% of respondents indicated that some countries and/or institutions do not have DTAs or MTAs in place to regulate the national or trans-border sharing of data. While MTAs were more common than DTAs, a fifth of the respondents were not even certain whether such transfer agreements existed within their affiliated institutions. Notably, although our findings indicate the absence of DTAs or MTAs at some institutions within SSA, most respondents (74%) indicated that their RECs were still responsible for reviewing these legal documents together with data sharing-related research protocols when required. This raises concern about the quality of review being conducted on the DTAs and MTAs submitted to RECs. Respondents perceived the development of comprehensive DTAs focused on safeguarding the privacy, anonymity and confidentiality of research participants as an effective resolution. Respondents emphasised that these DTAs should be stringent, with importance placed on institutions instigating mechanisms to improve regulatory compliance. Suggestions included consultation with legal experts in the development of new DTAs, or improvements to current DTAs to ensure that they are aligned to existing laws or regulations. The implementation of access control systems that concentrate on standard criteria for data use and propositions may reduce the likelihood of data misuse, and may legally complement data transfer across borders.

Some respondents were of the view that their country’s laws were fragmented and consequently exacerbated ethical challenges, thus needing to be harmonised. This was echoed in the responses indicating that data sharing for research could be better regulated both within their institutions (70%) and nationally (71%). Suggestions to develop policies with clear frameworks or stringent standard operating procedures on data sharing emerged, along with improving awareness and access to adequate training on protocol review, data sharing, processing and protection. Likewise, over a third of respondents were not aware of the restrictions placed on the trans-border flow of research data at their institutions.

Many challenges exist in data governance in SSA. The lack of legal and ethics expertise within RECs was recognised as a challenge in adequately reviewing research protocols that related to big data, research transfer agreements and in developing frameworks and policies. Some respondents reported that their institutions do not have ethics (11%) and regulatory (8%) guidance in place for the protection of research data or HBMs, whilst others reported being unsure about whether such ethics (14%) and regulatory (9%) guidance were utilised within their institutions. These findings are comparable with the systematic review conducted by Barchi and Little^28^, who found that 29 of the 49 SSA countries (59%) had some form of national ethics guidance. Barchi and Little concluded that SSA countries that still lacked regulatory guidance on research data or HBMs would require extensive health-system strengthening in ethics governance before they could be fully engaged in the modern research enterprise.^28^

Respondents reported the development of adequate legal frameworks or ethics guidance and policies for research data protection within their respective countries as a pressing challenge. A lack of resources was identified as a common reason for this as respondents expressed an increased need for resources, such as training, to efficiently develop and maintain legislative frameworks for data protection in SSA.

Although some of the epistemic gaps presented with RECs could be addressed, some of the committees’ responsibilities may be seen as falling outside their mandate and scope of function. This drew attention to the question of who should review such documents when an epistemological challenge exists amongst RECs. Some authors have argued that such responsibility is incompatible with RECs’ legislative oversight role and that a legal body is better suited to review such legal documents.^11^

The current lack of training available in the field of data science for REC members to better handle the ethical, legal and social implications of big data-related research highlights the need to proactively educate and train^26^ SSA research-based institutions to foster and empower the formation of DACs^13,35^. While most respondents confirmed that their institutions lacked DACs to handle data-related issues in research, such committees could play a significant role in the data governance ecosystem.^13,35^ The suggestion to form institutional DACs emerged from our study results; however, respondents also indicated that difficulty may be encountered in establishing these committees with members of sufficient and diverse knowledge, skills and experience.

Training needs were evident across the subcontinent. REC members recognised a deficit in their experience and expertise pertaining to the review of research protocols involving big data and related research transfer agreements. This is evident in the large cohort of respondents (64%) that were not often exposed to research protocols that related purely to large data sets or big data as they clearly indicated that the bulk of all research protocols reviewed did not relate to data sharing at all. This finding was further strengthened by the third (32%) of respondents in our study who explicitly stated that they had not received any training on reviewing protocols involving data use and data sharing. Interestingly, 23% of respondents expressed uncertainty on whether they engage with data sharing related research protocols as a result of not entirely understanding what data sharing and big data essentially encompass. This training deficit is not unique to SSA. Ferretti et al.^2^ found that REC members in Switzerland faced similar challenges in adequately reviewing protocols involving big data research due to an existing lack of expertise and experience in the field.^2,36^ In Australia, Pysar et al.^15^ revealed that genomic confidence scores in reviewing related research protocols were low amongst REC members that were less experienced, and had less exposure and training in the field. Hence, most participants (76%) in this study indicated that non-genetics experts that serve on RECs require additional training and/or resources on big data research. Equipping RECs with basic epistemological advantages, in the form of skills and knowledge in big data, would allow them to better fulfil their roles in effectively reviewing data-sharing protocols.

Pisa et al.^37^ proposed addressing funding issues, strengthening data management systems, providing training and conducting workshops to strengthen regulatory capacity. This will reduce and mitigate instances of data exploitation or harm encountered by research participants and data subjects.

### Study limitations

A notable limitation to be acknowledged when interpreting the results of this study is the predominance of responses from some SSA counties compared to other countries (indicated in Figure 1). This may be due to a higher number of RECs in these countries, more active research sites and the fact that it was easier to locate active email contacts from representatives of these SSA countries. These findings were also from a relatively small survey. Potential participants without reliable internet access may have been unintentionally excluded from participation given the internet-based nature of the survey. Because these results were confined to the SSA context, and 15 of the SSA countries did not participate in our survey, we may not have been able to represent the entire continuum of variability present within the SSA region. However, given the absence of empirical studies on the awareness and perspectives of REC members in SSA, these limitations do not pose a major threat to our survey’s exploratory aim. Our qualitative research may address some of these limitations and will be published separately.

Overall, our highest number of survey responses was obtained from the Democratic Republic of the Congo, Kenya, Mozambique, Nigeria, South Africa and Uganda. This may be because most of these countries (South Africa, Nigeria, Kenya and Uganda)^38^ are ranked as the most research-intense countries in SSA by research output in the fields of public health, and environmental and occupational health^39–41^. The increased research activities in these SSA countries may be associated with increased cross-border data transfer.

South Africa and Kenya are the most stringent in their data export protection. For data to be transferred out of these countries, the data transfer must be purposeful, consent must be obtained from data subjects, and the data processor must verify to the data commissioner that the third-party recipient’s jurisdiction is bound by appropriate safeguards for the security and protection of the data.^42^ Yet, our results did not entirely reflect this, as not all responses from Kenya appeared to be in agreement, indicating a divide. Likewise, a divide was observed in the aggregated results from Nigeria, although the country is very research active. This may be because the country’s moderately rigid data export protection does not require third-party recipients of data to be bound by adequate data protection laws or agreements in cases where consent is acquired, or where the transfer meets an exception.^29,38^ For South Africa, the highest-ranked SSA country by research output in public health, and environmental and occupational health^38^, our results reveal consensus amongst respondents regarding cross-border data transfers, which may be due to awareness of POPIA^29,43^.

## Conclusion

In this study, we intended to provide a broad overview of REC members’ awareness and perceptions on data governance in SSA and related legal and ethical challenges. Our results uncovered valuable insights and offer a novel contribution to the empirical literature in SSA on big data. Our findings indicate variability in data governance and regulation in SSA, as well as variability in REC members’ perceptions of the adequacy of their national laws and institutional policies. Suboptimal awareness of the existence of data protection laws or the lack thereof amongst REC members in the sample was concerning. This will impact negatively on how data-intense protocols are reviewed. There is a unanimous expressed need for the training of REC members on the African continent. Established RECs across SSA would benefit from the reformation of practices and oversight mechanisms, expertise and regulations to better cater for the big data research context. Transparent, robust and standardised data governance may promote shared ethical values to conduct research with big data on the subcontinent. Data governance within SSA continues to be inadequately supported by legislative and enforcement frameworks.

## Figures and Tables

**Table 1:** Characteristics of survey respondents (*N* = 140)

Characteristic	*n* (%)
**Gender**	
Male	88 (63)
Female	51 (36)
Other	1 (1)
**Education/qualification**	
Bachelor’s degree	7 (5)
Honours degree	6 (4)
Master’s degree	58 (41)
Doctoral degree	64 (46)
Other	5 (4)
**Type of REC**	
National	37 (26)
Institutional	96 (67)
Private	7 (5)
Position/role	
Chair	19 (14)
Co-chair	4 (3)
Vice-chair	5 (4)
Member	112 (80)
**Years of experience**	
Less than 2 years	20 (14)
2–4 years	19 (14)
4–6 years	29 (21)
6–8 years	13 (9)
8 or more years	59 (42)

**Table 2:** Respondents’ awareness of laws and policies on research data protection (*N* = 140)

Any law on research data protection in the country?	*n* (%)
Yes	82 (59)
No	24 (17)
Unsure	34 (24)
Restrictions/prohibitions placed on the trans-border flow of research data in the country?	
Yes	67 (48)
No	34 (24)
Unsure	39 (28)
Any policy on research data protection at the institution?	
Yes	96 (69)
No	26 (19)
Unsure	18 (13)
Restrictions/prohibitions placed on the trans-border flow of research data at the institution?	
Yes	70 (50)
No	48 (34)
Unsure	22 (16)

**Table 3:** Validation of responses received (*N* = 140)

Responses	Existing privacy laws		Total
	Yes	No	
Yes	67/107 (63%)	16/107 (15%)	83/107 (78%)
No	10/107 (9%)	14/107 (13%)	24/107 (22%)
Total	77/107 (72%)	30/107 (28%)	107 (100%)

**Table 4:** Respondents’ perceptions of data-related laws or policies (*N* = 140)

Adequate law on research data protection within the respondent’s country	*n* (%)
None (no law or policy)	25 (18)
Disagree strongly	12 (9)
Disagree somewhat	15 (11)
Unsure	25 (18)
Agree somewhat	46 (33)
Agree strongly	17 (12)
Adequate restrictions or prohibitions on the trans-border flow of research data at country level	
None (no law or policy)	16 (11)
Disagree strongly	17 (12)
Disagree somewhat	18 (13)
Unsure	33 (24)
Agree somewhat	46 (33)
Agree strongly	10 (7)
Adequate institutional-level policy on research data protection	
None (no law or policy)	17 (12)
Disagree strongly	8 (6)
Disagree somewhat	21 (15)
Unsure	22 (16)
Agree somewhat	54 (39)
Agree strongly	18 (13)
Adequate institutional-level restrictions or prohibitions on the trans-border flow of research data	
None (no law or policy)	22 (16)
Disagree strongly	12 (9)
Disagree somewhat	18 (13)
Unsure	31 (22)
Agree somewhat	46 (33)
Agree strongly	11 (8)

**Table 5:** Protection of research data or HBM (*N* = 140)

Separate DTA available at the respondent’s institution	*n* (%)
Yes	50 (36)
No	90 (64)
Separate MTA available at the respondent’s institution	
Yes	74 (53)
No	66 (47)
Combined DTA and MTA available at the respondent’s institution	
Yes	33 (24)
No	107 (76)
My institution has appropriate regulatory policies in place	
None	16 (11)
Disagree strongly	8 (6)
Disagree somewhat	6 (4)
Unsure	19 (14)
Agree somewhat	48 (34)
Agree strongly	43 (31)
My institution has appropriate ethics guidance in place	
None	11 (8)
Disagree strongly	3 (2)
Disagree somewhat	5 (4)
Unsure	12 (9)
Agree somewhat	44 (31)
Agree strongly	65 (46)

HBM, human biological material; DTA, Data Transfer Agreement; MTA, Material Transfer Agreement

## Significance:

This is the first empirical survey in SSA in which awareness and perspectives of REC members have been explored specifically relating to the review of data-intense research protocols. Big data have raised new ethics and legal challenges, and this survey provides a broad overview of these challenges in SSA. Our study confirms that knowledge and awareness of legislative frameworks and ethics guidance in SSA vary considerably where big data are concerned. The research results could be useful for a range of stakeholders, including RECs, data scientists, researchers, research and academic institutions, government decision-makers and artificial intelligence (AI) coders.
